# Hardware-Efficient Scheme for Trailer Robot Parking by Truck Robot in an Indoor Environment with Rendezvous

**DOI:** 10.3390/s23115097

**Published:** 2023-05-26

**Authors:** Divya Vani G, Srinivasa Rao Karumuri, Chinnaiah M C, Siew-Kei Lam, Janardhan Narambhatlu, Sanjay Dubey

**Affiliations:** 1 Department of Electronics and Communication Engineering, Koneru Lakshmaiah Education Foundation, Green Fields, Vaddeswaram 522502, India; 2 Department of Electronics and Communication Engineering, B V Raju Institute of Technology, Medak 502313, India; 3 School of Computer Science and Engineering, Nanyang Technological University, Singapore 639798, Singapore; 4 Department of Mechanical Engineering, Chaitanya Bharati Institute of Technology, Hyderabad 500075, India

**Keywords:** trailer robot, parking, rendezvous behavioral control, FPGA

## Abstract

Autonomous grounded vehicle-based social assistance/service robot parking in an indoor environment is an exciting challenge in urban cities. There are few efficient methods for parking multi-robot/agent teams in an unknown indoor environment. The primary objective of autonomous multi-robot/agent teams is to establish synchronization between them and to stay in behavioral control when static and when in motion. In this regard, the proposed hardware-efficient algorithm addresses the parking of a trailer (follower) robot in indoor environments by a truck (leader) robot with a rendezvous approach. In the process of parking, initial rendezvous behavioral control between the truck and trailer robots is established. Next, the parking space in the environment is estimated by the truck robot, and the trailer robot parks under the supervision of the truck robot. The proposed behavioral control mechanisms were executed between heterogenous-type computational-based robots. Optimized sensors were used for traversing and the execution of the parking methods. The truck robot leads, and the trailer robot mimics the actions in the execution of path planning and parking. The truck robot was integrated with FPGA (Xilinx Zynq XC7Z020-CLG484-1), and the trailer was integrated with Arduino UNO computing devices; this heterogenous modeling is adequate in the execution of trailer parking by a truck. The hardware schemes were developed using Verilog HDL for the FPGA (truck)-based robot and Python for the Arduino (trailer)-based robot.

## 1. Introduction

Developing an intelligent transportation system (ITS) has been a leading research area in the last two decades. There are various ITS approaches, such as an individual, multimedia, and goods-carrying ITS. The world market is dependent on export and import trading, and most shipments are carried out using an ITS type of truck–trailer. There are a lot of challenges in autonomous truck–trailer approaches. Trucks generate good revenue and tonnage. A study was conducted by the American Trucking Association (ATA) [1] regarding various challenges in transportation by road. Autonomous truck–trailer vehicle methods were discussed by Lai et al. [2], and they determined the error thresholds regarding vehicle dimensions. Autonomous vehicles/robots are unified by their sensing-, perception-, decision-, operating-system-, computational-device-, and application-based hardware platform [3]. In an autonomous robotic system, the decision structure consists of path planning, action prediction, obstacle avoidance, feedback control, and behavioral control.

Currently, autonomous cars/robots are becoming more popular in the market. An ITS mostly relies on three regions of research on truck–trailer systems, including the kinematics of truck–trailer balancing, road challenges, and the parking of the truck–trailer. The parking concept involves the integration of a few decision parameters, such as path planning and kinematics-based action prediction. The proposed approach is related to the parking of the truck–trailer method. Vital et al. [4] described intelligent truck parking issues and approaches, such as an increase in parking capacity, a decrease in peak demand, resource allocation using hours of service (HoS), scheduling and routing, and space occupancy detection. Pan He et al. [5] classified truck–trailer systems based on various aspects to estimate the size of the truck and trailer. A primary aspect of truck–trailer parking is the model and prediction with respect to the size of the truck–trailer while it performs the parking. In this regard, prior truck–trailer analysis is essential to observe the standard truck size and different sizes of trailers that are connected to trucks.

Autonomous path planning algorithms were developed by A. C. Manav et al. [6] regarding the iterative analysis method (IAM) and deterministic parking maneuvers. Parking is one of the major factors in ITS, which has been differentiated with respect to outdoor and indoor environments. In indoor environments, two means of parking are available, one for vehicles (cars) and the other for autonomous robots. Various smart parking assistance studies were performed by T. Lin et al. [7]. These studies were focused on the collecting of information, system deployment, and the service dissemination of autonomous parking. Y. Huang et al. [8] addressed parking in indoor environments using a vision-based semantic mapping approach. Assisting drivers to park trailers was discussed by V. Josef [9]. Ruofan Kong et al. [10] discussed truck–trailer mobile robot parking. In early 2020, D. J. Cook et al. [11] discussed truck–trailer freight hauling impacts on the environment, thus providing a vision-based automated solution.

Indoor service/social robots have been used as a major part of diversified applications. As per an indoor robot market analysis, the market was worth USD 11.65 billion in 2021 and will grow by 25% per year from the present status to USD 100.37 billion by 2029 [12]. Outdoor parking challenges have been discussed by W. Kim et al. [13], H. Banzhaf et al. [14], and N. Fulman et al. [15]. The parking of robots in an indoor environment also depends on the technology used for sensing the environment, calculating it, and collision avoidance. In this regard, we cannot incorporate all parking challenges and their solutions in every service/social robot; one service robot is enough to take care of sensing the environment and aligning itself properly with the social robots to park in the defined space unobstructed by living beings. Multi-robot approaches are trending with both centralized and distributed approaches, including the existing truck–trailer (physical interface) type. Rendezvous-based multi-robot coordination approaches have been discussed by researchers [16,17,18,19]. Recently, J. Yu et al. [20] addressed leader and follower robot exploration in environments using intra- and inter-behavioral senses. Multi-robots should be capable of sensing the environment, coordinating with other robots, and performing according to various decision parameters with an appropriate computation device, such as a field-programmable gate array (FPGA).

Autonomous parking in a real-time scenario depends on computational devices. In general, CPU/laptop/microprocessor devices are interfaced to robots/vehicles to perform parking. C. Huang et al. [21] developed vehicle parking guidance methods using a microprocessor to control the system. A microcontroller-based design is also used for computing parking, and it has been executed with a delayed approach in other devices. Naji, Baligh et al. [22] recently discussed the FPGA-based versatile parking of robots. S. Liu et al. [3], Z. Wan et al. [23,24], P. Vyas et al. [25], and Divya et al. [26] have determined that an FPGA is a pertinent computation device for autonomous vehicles and robotic applications. A recent study by S. Liu et al. determined that the cost of an autonomous vehicle/robot’s sensors and computational device was around USD 1,000,000 [27]. There is a tradeoff between the cost and precision in this approach, while achieving higher precision depends on vision-based approaches, such as SIFT, SURF [28], and ORB [29]. According to the observations from the above literature review, limited research on autonomous truck and trailer robot parking in indoor environments has been published. With reference to [7,8,9], parking with coordination between robots is challenging, and few researchers have addressed this concern [16,17,18,19]. The coordination of multiple robots is dependent on the computational device used to compute sensory information, behavioral control, and parking algorithms. The above methods described in [7,8,9] and [16,17,18,19] do not use parallel computing devices. In this regard, to overcome the above challenges in truck and trailer parking, a method is proposed using FPGA-based hardware schemes.

Higher-level FPGAs, such as Xilinx Kria or Zynq Ultra-Scale, are essential. With respect to affordability, the proposed approach is to integrate ultrasonic sensors with a low-cost FPGA (Xilinx Zynq XC7Z020-CLG484-1) to provide a solution for the parking of trailer robots for industry/household applications.

In this regard, the proposed approach is novel in the following ways:

1. A new behavioral control mechanism was developed with a rendezvous approach to perform parking by using a trailer robot with mimic methods.
2. The proposed hardware scheme is for the parking of a trailer robot under the leadership of an FPGA-based truck robot.
3. Slot identification by the leader (truck) robot which communicates it to the follower (trailer).

This paper is organized as follows. In this section, we have discussed the problems stated in the related literature and the originality of the proposed approach. We describe the methodology and hardware schemes for trailer robot parking by a truck robot in Section 2. Section 3 discusses the results, including a simulation, synthesis, and experimental validation. Finally, Section 4 presents the conclusion of this study.

## 2. Hardware-Scheme-Based Algorithm for Trailer Robot Parking

**Figure 1:**
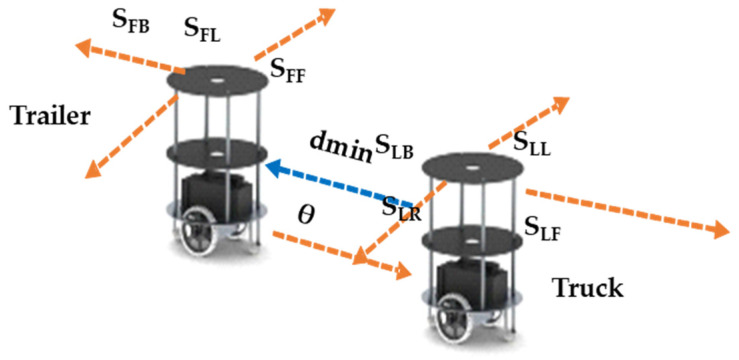
Truck and trailer robots in behavioral control and parking of trailer robot.

The proposed approach presents autonomous truck–trailer parking as part of intelligent transportation in the process of parking a trailer (follower) robot by a truck (leader) robot using a rendezvous methodology in an indoor environment, as shown in Figure 1. Truck robots have higher intelligence, and trailer robots follow the truck and mimic its activities. The steps of the process are as follows: (1) rendezvous behavioral control between the truck and trailer robots is established; (2) the parking space is estimated with sensory information; and (3) parking of the trailer robot is carried out using a mimic approach. The truck and trailer robots communicate with the rendezvous approach; however, they perform based on the leader-and-follower method.

### 2.1. Methodology of Trailer Robot Parking

#### 2.1.1. Behavioral Control between Truck and Trailer Robots with Rendezvous

**Figure 2:**
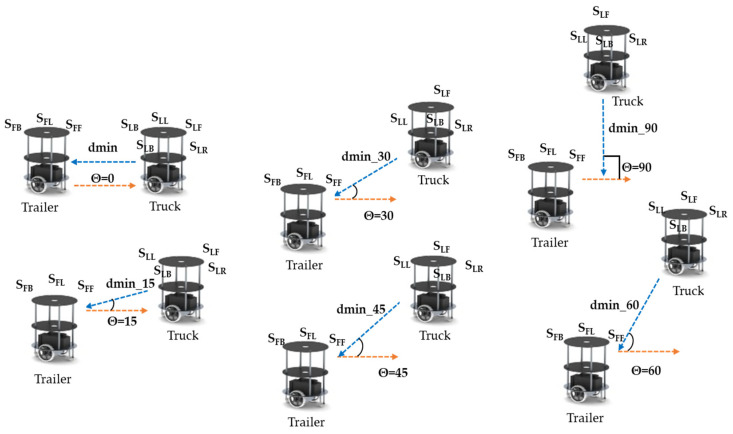
Hitch and side-slip angles between the truck and trailer robot.

Behavioral control between a truck and a trailer is established as the truck robot evaluates the trailer robot’s movements based on its hitch angle, side-slip angle, and velocity movements, which are represented in Figure 2. However, the current hitch angle between the truck and trailer is essential to performing the next level of robotic algorithms, including slot identification and mimic-based parking, as illustrated in Figure 2. When a robot is off of the hitch angle, it is in a position with various side-slip angles at any time in the process of execution. In this regard, alignment correction between the truck and trailer robots is performed using a side-slip angle approach. Behavioral control is performed with optimized sensors, which impacts the device computation, draining the battery.

#### 2.1.2. Hitch and Side-Slip Angle of Trailer Robot Evaluated by Truck Robot

The trailer robot is placed behind the truck robot, one ultrasonic sensor (S_LB_) is placed at the back of the truck robot, and a pair of infrared sensors are deployed at both ends of the robot (I_LBL_ and I_LBR_). Similarly, one ultrasonic sensor (S_FF_) and a pair of infrared (I_FFL_ and I_FFR_) sensors are placed on the front of the trailer robot. As shown in the current algorithm, line 3 represents the hitch angle analysis, which indicates if the truck and trailer are aligned, represented in line 6. If they are not aligned properly, then the side-slip angle variance between the robots is estimated, as shown in Algorithm 1 in lines 4 and 5 and illustrated in Figure 2. The truck robot analyzes the side-slip and performs the corrections, and at the same time, the trailer mimics the truck’s activities.
**Algorithm 1:** Pseudocode for rendezvous behavioral control between truck and trailer1.  Initialize, dmin_ ϑ {0,15,30,45,60,75,90,135}, 2.  Case (state) 3.  Bhv1: ((S_LB_, I_LBL_, I_LBR_) && (S_FF_, I_FFL_, I_FFR_) @ dmin_ϑ_0_^0^)? Bhv4: Bhv2 4.  Bhv2: if ((S_LB_, I_LBR_) > dmin_ϑ_0_^0^& ((S_FF_, I_FFL_) < dmin_ϑ_0_^0^& I_FFR_ > dmin_ϑ_0_^0^)            & (S_LL_ = dmin_ (−ϑ_90_^0^)))             {T_L_ wait, T_F_ rotate @ left_ϑ_15_^0^}       Else         {T_L_ wait, T_F_ rotate @ right_ϑ_15_^0^} 5. Bhv3: (S_LL_ = dmin_(−ϑ_75_^0^, −ϑ_60_^0^, −ϑ_45_^0^)) ?    {T_L_ wait, T_F_ @ left_ϑ_30_^0^, ϑ_45_^0^, ϑ_60_^0^}: {T_L_ wait, T_F_ @ right_ ϑ_30_^0^, ϑ_45_^0^, ϑ_60_^0^} 6.  Bhv4: If ((S_LB_ && S_FF_) @ dmin_ϑ_0_^0^) && ((S_FF_) @ frequency))         {T_L_& T_F_ forward @ dmin}       Else         {T_L_ forward@ T_F_ frequency} 7. end case

#### 2.1.3. Velocity Movement Control between Truck and Trailer Robots

While the alignment of the truck and trailer robots is achieved by traversing from one point to another point, the velocity between the robots is the most important parameter to avoid a collision and sliding from the original position. The d_min_ value is evaluated by both robots’ sensors (S_LB_, S_FF_). As illustrated in Figure 2, the truck and trailer robots move at different speeds; the trailer has a fixed speed, but the truck (T_L_) will slow down if it is moving faster than the trailer (T_F_). Similarly, when the T_L_ is slower than the T_F_, it will speed up. This is shown in the algorithm in line 6 regarding the velocity control mechanism.

#### 2.1.4. Parking Slot Identification with Sensory Information

Truck–trailer robots can traverse in any direction in an indoor environment. Slot identification depends on the direction of the truck and trailer and the slot type. Parking slot types are classified into two categories: (1) a single-boundary-based slot and (2) a double-boundary-based slot. In indoor environments, at certain times, an exact parking slot cannot be found, and robots must define a parking space based on a single-reference boundary, which is referred to here as a single-boundary-based slot. A double-boundary-based slot is (a) between two static boundaries provided for a parking slot, where one is at the edge of the environment and the other is a parking boundary or another parked vehicle, or (b) a semi-dynamic parking slot between two vehicles/robots.

**Figure 3:**
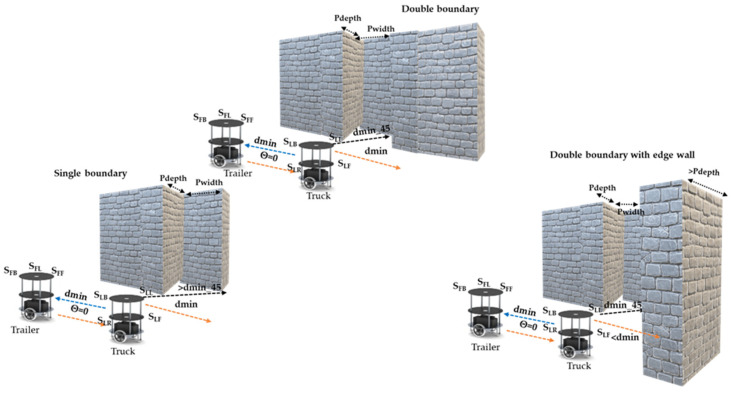
Parking slot identification by truck–trailer robot.

The truck (T_L_) robot performs slot identification and recommends it to the trailer (T_F_) robot to perform the parking. In this regard, the dmin values are evaluated for various angles, as shown in Algorithm 2 (slot identification by the truck robot) in line 1. The truck (T_L_) robot’s front sensor (S_LF_) evaluates the distance and finds a free space to traverse in the next level of navigation, with the left side sensor of the T_L_ (S_LL_15_) at an angle of ϑ_15_^0^, which is shown in Figure 3. When it is within the range, the robot performs a forward action (lot2), as defined in line 3. Line 4 represents sufficient parking space determined using the T_L_ left sensor (S_LL_135_) and front sensor (S_LF_45_). Line 5 classifies the respective parking slots. A double-boundary parking slot is between two static boundaries or edges, as shown in lines 7 to 8. If there is only one edge or boundary in the environment and the other side is open, it is considered a single-boundary-based slot, as illustrated in Figure 3 and defined in line 6 as well as in line 10 under different conditions. In certain scenarios, a slot is not available or is preoccupied by other vehicles/robots, as shown in line 11.
**Algorithm 2:** Pseudocode for parking slot identification by truck robot1. Initialize, dmin_ ϑ {0,15,30,45,60,75}, 2. Case (state) 3. lot1: (S_LL_15_ ≥ dmin_ϑ_15_^0^)? Lot2: lot4 4. lot2: ((S_LL_135_ ≥ dmin_ϑ_45_^0^) && (S_LF_(45)_ ≥ dmin_ϑ_45_^0^)) ? lot21: lot3 5.  Case (lot21) 6.  Lot_1: ((S_LL_90_ ≥ dmin_ϑ_90_^0^) && (S_LF_45_ ≥ dmin_ϑ_45_^0^))? Park_sb: Lot_2 7.  Lot_2: ((S_LL_90_ ≥ dmin_ϑ_90_^0^) && (S_LF_<dmin))? Park_edge: Lot_3 8.  Lot_3: ((S_LL_90_ ≥ dmin_ϑ_90_^0^) && (S_LF___45_ = dmin_ϑ_45_^0^))? Park_db: Lot_1 9.  end case 10. lot3: ((S_LL_90_ ≥ dmin_ϑ_90_^0^) && (S_LF_ (45)_ ≥ dmin_ϑ_45_^0^))? Park_sb: lot4 11. lot4: (S_LL_15_< dmin_ϑ_15_^0^)? Forward: lot1 12. end case

#### 2.1.5. Parking of Truck–Trailer Robot with the Mimic Method

The principle of the proposed method is the parking of a trailer robot by a truck robot using the mimic-based rendezvous approach. Previously, performing parking depended on the behavioral control between the robots and slot identification by the truck robot in various areas, such as those with a single boundary, a double boundary with a static boundary or edge, and a dynamic boundary.

**Figure 4:**
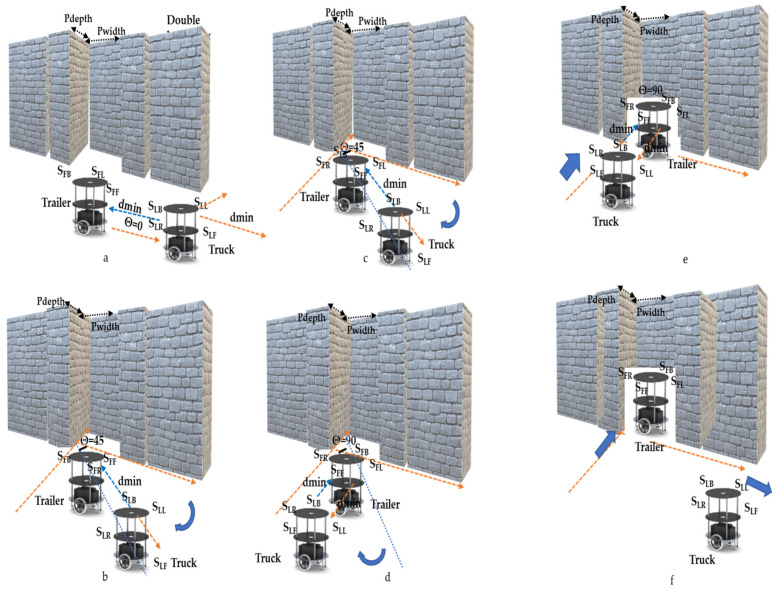
(**a**–**f**) Illustration of truck–trailer robot parking.

When truck–trailer robots are operating in a rendezvous mode, parking is performed along two pathways—feed-forward and return/reverse control. Once a slot is confirmed by the truck robot in feed-forward, the parking is performed within the single and double boundaries, as shown in Algorithm 3, lines 9–14. When a parking slot consists of an edge, parking is carried out using lines 15–20. As shown in Figure 4, a total of five levels of kinematic operation are performed during the execution of the parking algorithm by a truck–trailer robot. In level 1, the T_L_ takes the proper position, and it properly aligns the T_F_ with the parking slot. In level 2, the T_L_ performs angular movements and at the same time waits for the T_F_ to mimic it, as shown in lines 10 and 17 (Figure 4b–d). In the next level, the T_L_ moves towards the T_F_, and it understands the truck movements and mimics it is using hitch angles and velocity modules to park exactly in the respective position, as described in lines 11 and 19 (Figure 4e). As per the edge-based boundary conditions, extra steps are performed with the side-slip angles, as shown in line 18. Once the truck completes the trailer parking, it implicitly communicates that it is exiting the rendezvous (line 13) (Figure 4f) while moving along a reverse pathway (line 23). Similar to the feed-forward mode, the trailer robot also mimics the reverse algorithm steps. It drives the T_F_ to exit the parking slot, and in this process, forward and side-slip angular movements are performed (lines 24–26).
**Algorithm 3:** Pseudocode for parking by truck–trailer robotIf ((T_L_ & T_F_ = dmin) @ (S_LF_90_ ≥ dmin_ϑ_90_^0^) && (S_LL_(135)_ ≥ dmin_ϑ_45_^0^))forwardelsereturnendCase (forward)Park_pr: (S_LF_ ≥ dmin)? Park_db: Park_db1Park_db: (S_LF_45_ ≥ dmin_ϑ_45_^0^) && (S_LL_(135)_ ≥ dmin_ϑ_45_^0^)? Park_db1: Park_sbPark_sb: *Case (Park_Move)*Park_1: T_L_ Angle_move & T_F_ mimic @ (ϑ_15_^0^,_30_^,0^,_30_^0^,_15_^0^)Park_2: T_L_& T_F_ Move back @ behavioral control.Park_3: ((S_LL_135_ = dmin_ϑ_15_^0^) && (S_LR_(0)_ ≥ dmin_ϑ_90_^0^))? Stop: ParPark_4: T_L_ forward & back with dmin and leave parking point.end caseCase (Park_db1)Park_11: (S_LF_ ≥ dmin)? Park_1: Park12Park_12: T_L_ Angle_move & T_F_ mimic @ (ϑ_45_^0^)Park_13: T_L_ = dmin @forward, T_F_ mimicPark_14: T_L_ Angle_move & T_F_ mimic @ (ϑ_45_^0^), Parking_moveend caseend caseCase (return)Park_r1: T_L_ forward & back with dmin and T_F_ starts mimic.Park_r2: T_L_ & T_F_ Move forward @ behavioral control.Park_r3: T_L_ Angle_move & T_F_ mimic @ (ϑ_15_^0^,_30_^,0^,_30_^0^,_15_^0^)Park_r4: T_L_ & T_F_ Move forward @ behavioral control.end case

### 2.2. Hardware Schemes for Trailer Parking by Truck Robot

**Figure 5:**
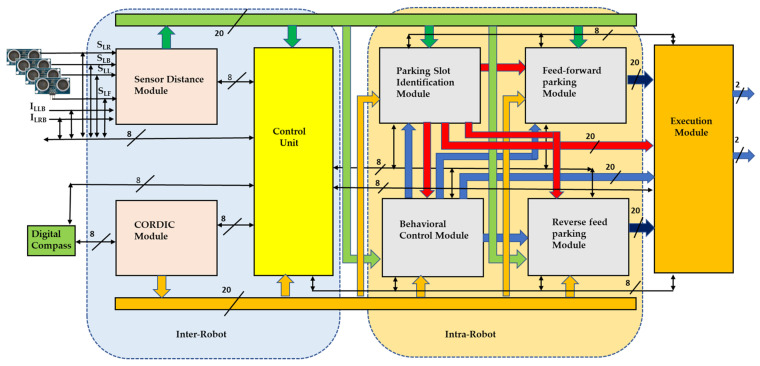
Architecture of truck robot.

This section presents intuitive hardware schemes, which allow efficient parking in an indoor environment. Subsequently, the novelty of the proposed development of hardware-based algorithms and their equivalents are presented in this section. Reliable and efficient trailer parking with a rendezvous approach by a truck robot is the main building block of parking. The building block consists of a processing module (PM) and an execution module (EM), and the PM consists of six limbs: (a) sensor distance module; (b) CORDIC module; (c) control unit; (d) behavioral control module; (e) parking slot identification; and (f) feed-forward and reverse parking modules, as presented in Figure 5. The truck robot unloads the trailer robot to perform parking, and the trailer robot follows the truck and mimics its respective activities. As shown in Figure 5, the trailer robot consists of inter-robot modules, such as a control unit, a sensor distance module, input sensors, and an execution module, and the intra-robot consists only of a behavioral control module (mimic module).

Most of the parking is performed by the truck robot, in which PWM ultrasonic sensors are interfaced to a field-programmable gate array (FPGA). It operates at 40 kHz with an echo-based approach. The received signal is in the pulse width modulation (PWM), and these data are converted into distances, which are transmitted to the main modules for parking. A coordinate rotation digital computer (CORDIC) with a Xilinx IP core is used to estimate the angles and the robot’s rotation, and the angle movements regularly depend on the CORDIC module. The CORDIC module is used to perform a square root to estimate the Euclidean distance from the domain at various angles (ϑ {0,15,30,45,60,75,90,135}).

In the process of parking execution, both robots are dependent on behavioral control, the hardware scheme of which is presented in Figure 5. Real-time sensor data (I_LBL_, I_LBR_, S_LB_, S_LL_, S_LR_, and S_LF_) are used to compute the orientation between the T_L_ and T_F_, as shown in the behavioral control module in Figure 5. This ensures that both the T_L_ and T_F_ operate at the same speed and perform in feed-forward motion as the Bhv1 output. Of course, the T_L_ and T_F_ are at a hitch angle, but different velocities are observed, in response to which the truck robot synchronizes its speed with the trailer robot by sampling the frequency with the clock divider circuits and resulting in the Bhv4 output. The Bhv2 output is driven by the side-slip angle between the T_L_ and T_F_ and represents the rotation of the angle ϑ_15_^0^; this module is regularly used while parking. Bhv2 is used in the process of gradual step movement. Subsequently, if the side-slip angles are higher than in Bhv2, Bhv3 is recommended, which will compute for angles ϑ {30,45,60,75,90,135}. An internal look-up table (LUT) is established for a quick response to compare with real-time conditions, and then the execution unit performs the operation.

**Figure 6:**
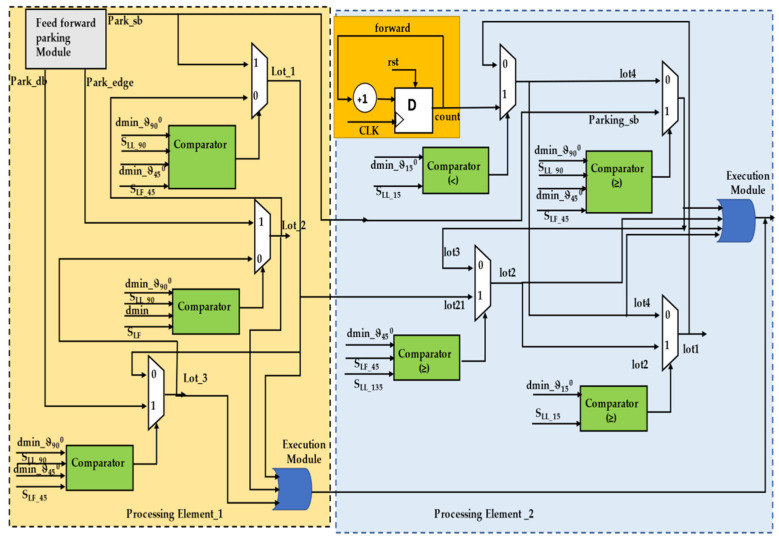
Internal hardware scheme of parking slot identification module.

The hardware schemes of parking slot identification are presented in Figure 6. Initially, the behavioral control module is applied to execute slot identification. The hardware scheme is mainly composed of four sub-modules, of which slot identification is the first and determines whether the depth and width of the slot is sufficient for robot positioning. The lot1-output-driven module performs forward action until it finds the best slot for parking. Once a parking space is close to the requirements, the next module is triggered. The second sub-module evaluates whether the parking slot has a double boundary and whether one side is an edge or a boundary, which impacts the performance of parking. The sensory data are key players in defining the internal states of the lot2 output. If the double-boundary conditions are not satisfied, the single-boundary slot evaluator module is triggered, which results in the lot3 output. The default module continues until it finds the exact slot specifications, which leads to the lot4 output.

**Figure 7:**
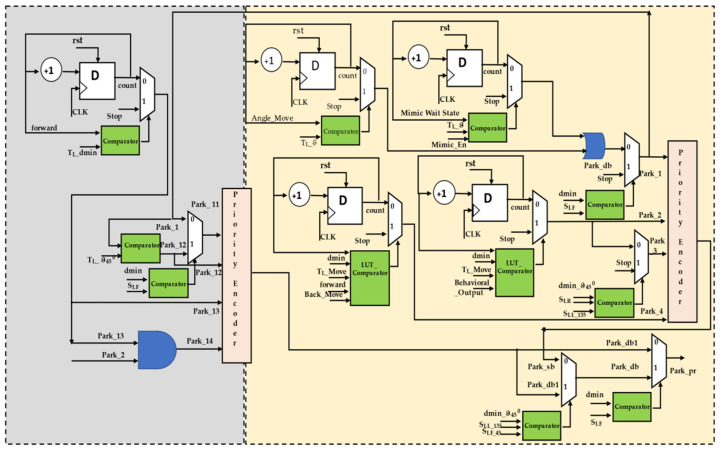
Internal hardware scheme for trailer parking by truck robot.

In this section, we describe how the parking of the trailer robot is organized by the truck robot, and the hardware schemes are shown in Figure 7. The slot identification and behavioral control modules transmit their evaluations to the parking module. Both the T_L_ and T_F_ are aligned based on slot availability, and parking is carried out based on the type of slot available. Two types of parking actions can be carried out: feed-forward and reverse. While executing feed-forward motion, the parking slot should be empty or occupied by the trailer robot with a behavioral control mechanism that ensures the truck robot pulls the trailer out of the parking slot according to the evaluation during the slot identification. While performing forward parking consists of processing elements for single-boundary parking, double-boundary parking consists of two boundaries or one boundary and one edge, where one boundary/edge is static and the other is dynamic (vehicle movements). The single-boundary module consists of four levels of hardware sub-modules, which are subtitled park_1 and consist of two counter modules, one for performing the angles of the T_L_ with respect to the conditions and the other to delay the T_L_ until the T_F_ mimics its activity. The angular movement conditions are compared between digital compass 32-bit information versus 32-bit CORDIC-generated angles as the reference movements required. The look-up table (LUT) influences the next level of operation of the single-boundary parking behavioral control. Before the final stage of parking, the T_L_ checks whether the trailer robot is parked exactly in the parking slot, which is continuously verified with sensory information, and it aligns it properly. In the final stage of single-boundary parking, the truck will move ahead two steps and back two steps twice; these movement-based steps are counter-designed, and the T_F_ will sense that the T_L_ is moving away from the behavioral control.

Subsequently, double boundaries have an approach like single-boundary parking, but this varies with respect to the edge/boundary/dynamic boundary. With a dynamic boundary, T_L_ recommends the single-boundary approach but with different conditions. When parking in a slot where both boundaries are static and without edge conditions, the T_L_ executes angular movements and waits for the T_F_ to mimic them (the T_L_ waits for the delayed counter design), driving the T_F_ into place in the parking slot. There is a different combination for parking in boundaries where one is an edge, and the T_L_ performs this parking with four sub-modules. In the first step, the T_L_ checks the sensory information to determine if the S_LF_ distances are less than dmin away, and then it moves to the right at an angle of ϑ_45_^0^ when the parking slot is on the left side of the T_L_. Similarly, when the slot is on the right side of the T_L_, it moves to the left. In the next step, until the angle is obtained, the angular counter causes the T_L_ to operate along the same line and continuously compares the reference value as per the algorithm and the real-time T_L_ angle. Next, the delay counter of the T_L_ runs in parallel until the T_F_ mimics the activity. Once this is accomplished, it continues to the next step in the algorithm and moves a few steps forward with respect to the conditions. In the pre-final stage, the T_L_ executes the angles and drives to the hitch angle from the side-slip angle. In the final stage, the aligned T_L_ and T_F_ move toward the parking space, and the T_L_ detaches from the T_F_, as mentioned above in the single-boundary parking approach. The Bluetooth HC05 is interfaced with the execution module, and after the accomplishment of the behavioral steps by T_L_ and T_F_, the same is communicated between them.

The reverse parking approach is the inverse of the feed-forward operation, as mentioned above. Once the T_L_ takes the correct position in front of the T_F_ using the sensory information, the T_L_ is an individual, and in this regard it quickly positions itself to define the space. The initial stage of the reverse parking imitation module internally consists of a counter design to execute the forward and backward movements twice by maintaining the minimum distance between the T_L_ and T_F_ based on their behavior coordination. In this process, the back-to-back T_L_ and T_F_ move forward until the T_F_ is outside of the boundaries of the parking space. The real-time distances are compared using the comparators, and the velocity control module is utilized to synchronize the robots. In the next step, the T_L_ performs angular movements and waits for the T_F_ to mimic its respective activity, for which the counter and comparator designs are used. Based on the decision of the T_L_ requirements to move in either direction, the T_F_ mimics the respective operations of the behavioral control module.

The execution module consists of the motor control logic, the velocity module, and their respective signals that are transmitted to the motor driver circuits. In this regard, the speed control and forward, angular, and reverse (backward) movements of the motors are incorporated in this module.

## 3. Results

**Figure 8:**
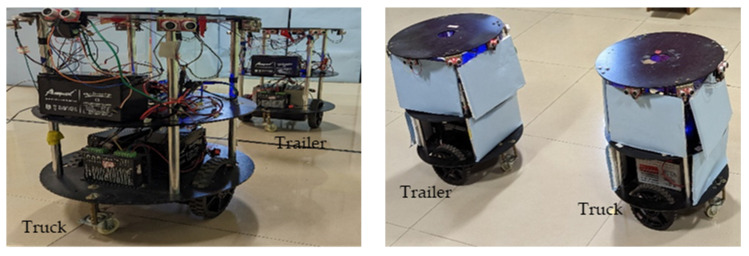
Truck–trailer mobile robot experimental setup.

In this section, we present a simulation and the experimental results of parking a trailer (follower) robot in an indoor environment by a truck (leader) robot using the rendezvous approach. The mobile robots were fabricated locally, as shown in Figure 8.

### 3.1. Experimental Setup

The experimental validation of the proposed hardware-efficient schemes was carried out using mobile robots. The hardware-efficient schemes were coded using Verilog HDL for the truck robot, which were simulated, synthesized, and implemented using an FPGA (Zynq XC7Z020-CLG484-1). The trailer robot control module was implemented using the Arduino module. It performed the mimicked actions by observing the truck robot.

The robots in Figure 8, constructed with 2 driving wheels that were 10 cm in diameter, were positioned in the middle of the platform. The free wheels were placed at the front end and back end of the platform. The robot platform was 5 cm above ground level, and each wheel was interfaced with a 20 kg torque stepper motor. The stepper motor driver SEA5045 module bridged the motors and the computational device. The mobile robots were powered by 24 v, 7 A lead–acid batteries. The computational devices were interfaced with a pair of stepper motors through driver modules and operated via the execution module of the hardware scheme, which consisted of essential motor control logic. The mobile robots were interfaced with Hitech HS-55 servo motors and a set of 4 PWM-type ultrasonic sensors and operated at a 20 KHz frequency and triggered by computational devices. The PWM echo signals were digitized (20 bit) and converted into distances. These distances played a vital role in the computation of the hardware schemes. The other interfaced sensors were a pair of IR sensors and an LSM303DLH-type digital compass tilt module. The digital compass was interfaced with the I^2^C protocol and operated using the CORDIC IP with a Xilinx core. For communication between the truck and trailer mobile robots, one used an ultrasonic sensor, and the other used a Bluetooth module. The ultrasonic sensors regularly estimated the movement of the rendezvous team member, and the Bluetooth module HC05 was interfaced using the UART protocol. This used an intercommunication approach after the accomplishment of each of the behavioral control steps.

#### Simulation Results of Behavioral Control between Truck and Trailer Robots with Rendezvous

**Figure 9:**
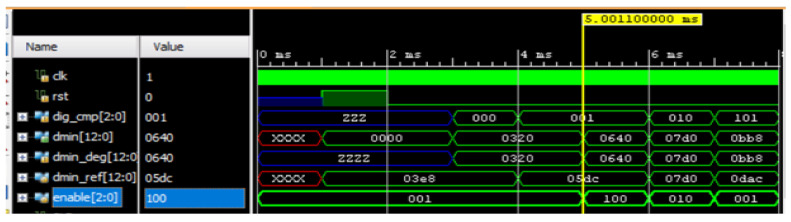
Simulation results of rendezvous behavioral control mechanism between truck and trailer robots.

Figure 9 illustrates the simulation results, showing how the truck robot carried out the behavioral control between the truck and trailer robots. The dmin_ref represents the action value as per the algorithm; real-time sensory information was used to evaluate the dmin value. The dig_cmp [2:0] represents the direction of the truck robot. At the time of 5.00 ms of the simulation, the dig_cmp was 3′b001, which indicates that the truck robot was at 15°, and the distance between the truck and the trailer robots was read by dmin [12:0]. The hardware scheme evaluated the velocity and minimum distance between the robots using dmin [12:0]. The dmin_ref values for 15° were compared with the distance obtained by the truck robot, and the enable [2:0] evaluated whether the distance between them was in alignment or not. In the process of the rendezvous approach, the velocity of the robots was evaluated using dmin. The comparator module output was called enable, and it determined if both robots were aligned with the line. A higher deviation was represented with enable [2:0] as 100 and a lower deviation as 001. The internal hardware scheme aligned the robot position to reach the balancing position as per the algorithm, and then the robots were aligned, which was represented with the signal enable [2:0] as 010.

### 3.2. Experimental Results

#### 3.2.1. Parking Slot Identification by Truck Robot with Behavioral Control Mechanism

**Figure 10:**
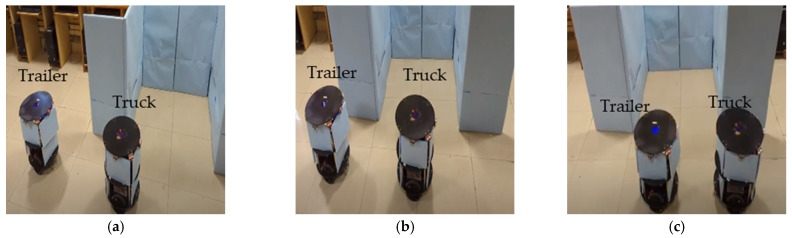
(**a**–**c**) Experimental results of parking slot identification by truck robot.

Figure 10a–c demonstrate the experimentation of the slot identification by the truck robot with rendezvous. As shown in the algorithm and hardware schemes, when using the rendezvous approach, the truck robot takes the lead and finds the parking slot. The minimum distance (dmin) between the robots was maintained while performing the slot identification. As presented in Figure 10a–c, in sequence level 1, the truck analyzed the space with the front and left sensors placed on the front side of the robot and maintained a parallel rendezvous with the trailer.

At level 2, both robots performed at the same velocity, and the truck evaluated the middle of the parking slot. The truck directed the trailer into place in the landmark, which was previously evaluated. If there was not sufficient space for the robot to park, the rendezvous team moved away from this slot.

#### 3.2.2. Trailer Robot Parking with Mimic Method

**Figure 11:**
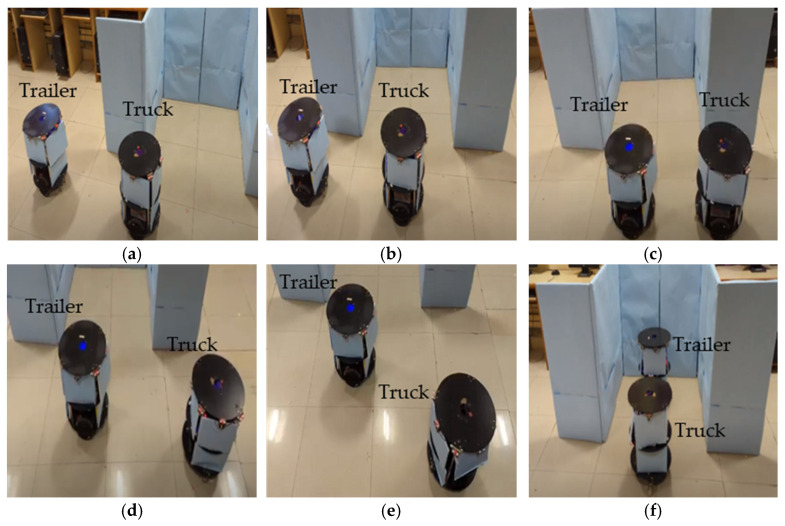
(**a**–**f**) Experimental results of trailer robot parking by truck robot.

**Figure 12:**
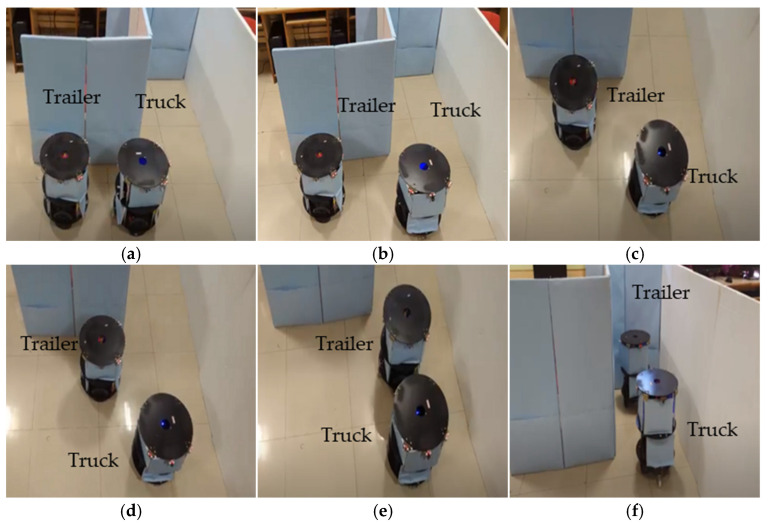
(**a**–**f**) Experimental results of trailer robot parking by truck robot at boundary with edge conditions.

Figure 11a–f demonstrate the experimentation of the double-boundary-based trailer parking by the truck robot using the mimic method. Once the slot identification was accomplished, the truck robot performed an angular movement with an equivalent distance movement. The trailer performed the angular operation and was evaluated as either aligned with the truck robot or with alignment errors, as self-analyzed by each robot using the sensory feedback mechanism, in which case a correction was performed. As per the algorithm, a sequence of small steps (15°, 30°, 30°, 15°) was used to reach an angle of 90° from the current position of the trailer. The truck adopted the waiting state until the trailer reached its position. Once both robots were completely aligned with the parking space, the truck took steps towards the trailer, and the trailer took steps towards the parking space. The truck continued this process until the trailer was in the parking space, and the parallel truck evaluated the status of the parking using the sensory information. The double-boundary approach came into the picture while parking the trailer with reference to the boundaries; the truck maintained the trailer parking movements without colliding into the boundaries of the environment. Similarly, Figure 12a–f present the scenario of one boundary and one edge or border. The angular movements of a robot team parking between a boundary and an edge are different compared with parking between a double boundary, as shown in the parking algorithm.

After the successful achievement of the trailer robot parking, the truck detached from the trailer until the next stage of operations. Hence, the truck robot conformed to the trailer, leaving it in the parking space using the Bluetooth module. With this movement, the trailer robot understood that the truck robot was disconnecting from the virtual connection. In the next stage, reverse parking was performed by the team, and similar steps were performed by the truck with signal movements, establishing communication between the robots.

#### 3.2.3. Trailer Robot Parking in Single-Boundary Conditions

**Figure 13:**
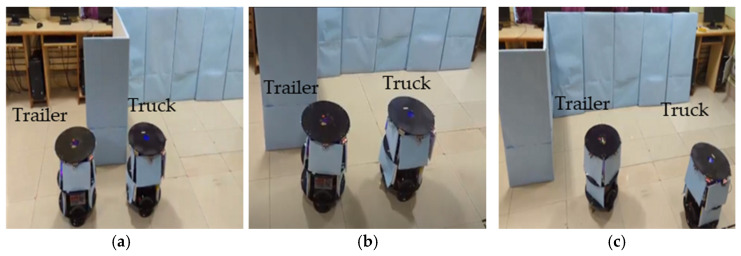

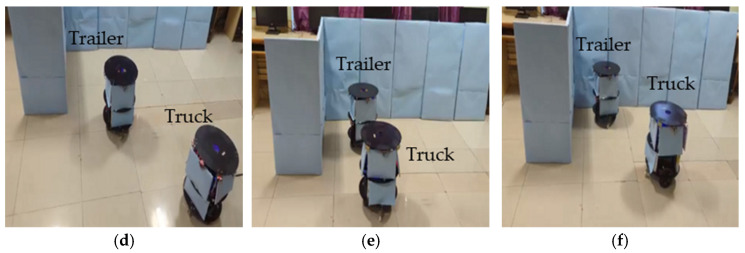
(**a**–**f**) Experimental results of trailer robot parking by truck robot in single-boundary conditions.

The single-boundary-based parking trailer robot is a novel parking approach, as demonstrated in Figure 13a–f. The truck robot determined the boundaries and space availability and drove the trailer robot into the slot with respect to the single boundary, with open space on the other side. The boundary was the reference mark, so the sensory information of the truck and trailer robots was different. Based on the conditions, the truck made sure the trailer was placed in the appropriate space without colliding with anything in the environment. The trailer was placed using the perfect geometry method in the next step so that if the truck wanted to place other robots it could place them beside the parked trailer robot in a similar fashion. The trailer robot performed the single-boundary parking using the mimic method until the parking was complete. Once the parking was accomplished, the truck robot detached from the trailer like in the above-mentioned double-boundary methods.

**Figure 14:**
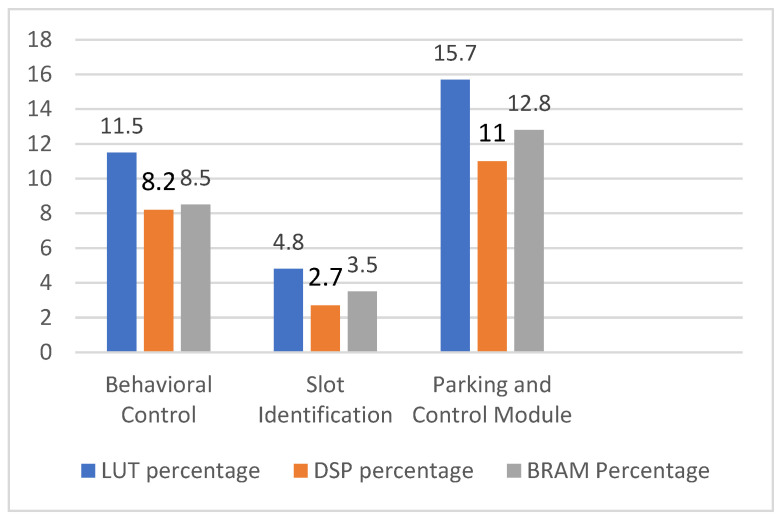
Device utilization of trailer robot parking by truck robot.

The proposed hardware schemes were simulated and synthesized; Table 1 represents the device utilization of FPGA resources. The overall device utilization of the resources was as follows: 32% LUTs, 25% BRAM, and 22% DSP slices of the FPGA. Individual modules of the proposed hardware schemes were deployed in Zynq XC7Z020-CLG484-1. The resource utilization is illustrated in Figure 14. During the research stage of the present contribution, the proposed approach was compared with other methods, as shown in Table 2. The comparison was conducted based on the sensor technologies, methodology, and computation devices. Computation devices play a vital role in the methodology for parallel computing with lower power consumption and effective timing. In this regard, an FPGA computation device is preferred over other computation devices such as CPUs and microcontrollers. The proposed method is a novel approach in truck–trailer robot parking in indoor environments that is comparable to [22]. In terms of computation, the proposed approach used FPGA-based computation technologies to achieve better results than [30,31,32,33].

## 4. Conclusions

In this article, we propose hardware-efficient schemes for trailer robot parking led by a truck robot that can be used in indoor-environment-based service/social robotic applications. The hardware-based algorithms and their equivalent reconfigurable architectures supported the implementation of trailer robot parking. We propose a novel hardware-efficient scheme for trailer robot parking with a truck robot that is able to carry out slot identification and places the trailer in an appropriate parking place, with a mimic-based behavioral control mechanism for different parking spaces. The proposed hardware schemes for the leader (truck) robot were coded in Verilog HDL and implemented using a reconfigurable FPGA device (Xilinx Zynq XC7Z020-CLG484-1). The follower (trailer) robot was interfaced with Arduino and mimicked the leader robot’s activities. Overall, the devices used for this approach consisted of 32% LUTs, 25% BRAM, and 22% DSP slices of the FPGA. In the future, the proposed concept will be implemented with a partial reconfiguration in a large indoor environment.

## Figures and Tables

**Table 1:** Utilization of proposed trailer robot parking.

Modules	LUT	FF	DSP	BRAM
Behavioral control and device interfacings	6128	12,256	18	12
Slot identification	2554	5108	6	5
Parking and control module	8342	16,684	24	18

**Table 2:** Comparison of different parking approaches.

Authors	Sensing Technologies	Methodology	Computation Device	Advantages	Remarks
Bing Li et al. [30]	LIDAR-based odometer and camera	Parking in garage using multi-story approach	CPU	Local SLAM has been executed in a shopping mall.	Limited to individual parking
Jiren Zhang et al. [31]	Integrated ultrasonic and LIDAR technologies	S-shape-based parallel parking executed	CPU	Real-time implementation in indoor environment.	Limited to individual parking
Jungwook Han et al. [32]	LIDAR	Perpendicular parking	NI Compact RIO (FPGA)	Indoor map building for parking.	Limited to individual parking
Deniz Ozsoyeller et al. [17]	Not available	Online-based mobile robot rendezvous	CPU	Multi-robot rendezvous with simulation results.	Limited to simulation
Bai Li et al. [33]	Not available	Optimal control-based parking approach	CPU	Framework and map developed based on lightweight methods.	Limited to individual parking
B. Naji et al. [22]	IR sensors	Both angular and parallel parking methods mentioned	FPGA	Versatile parking using an FPGA-based robot.	Limited to individual parking with simulation results
Proposed truck–trailer robot parking	Ultrasonic sensor and IR sensor	Mimic-based trailer robot parking in lines with perpendicular approach using rendezvous	FPGA	Hardware-efficient schemes for truck–trailer robot parking with rendezvous.	In future, partial reconfiguration approach integration will decrease device utilization

## Data Availability

The data presented in this study are available in Supplementary Materials.

## Supplementary Materials

The following supporting information can be downloaded at: Trailer mobile robot parking under truck robot guidance at double boundary conditions https://www.youtube.com/watch?v=AsvuiKTyhGw. Trailer mobile robot parking under truck robot guidance at Single side boundary conditions https://www.youtube.com/watch?v=TrrhC4qkuc0. Trailer mobile robot parking under truck robot guidance, while parking is between boundary and wall in an Indoor environment https://www.youtube.com/watch?v=tWQCjtBJblg&t=5s. Trailer mobile robot traversed out from parking under the leadership of truck robot, https://www.youtube.com/watch?v=ll8eA4pZKJI.

## Abbreviations

**Abbreviation**	**Expansion**
dmin_ϑ	Minimum distance of the sensor.
S_LB_ ϑ_	Back sensor of leader (Truck) robot at an angle of ϑ.
I_LBL_ ϑ_	Left corner IR sensor in Back side of Leader (Truck) robot at an angle of ϑ.
I_LBR_ ϑ_	Right corner IR sensor in Back side of Leader (Truck) robot at an angle of ϑ.
S_FF_ ϑ_	Front sensor of Follower (Trailer) robot at an angle of ϑ.
I_FFL_ ϑ_	Left corner IR sensor in Front side of Follower (Trailer) robot at an angle of ϑ.
I_FFR_ ϑ_	Right corner IR sensor in Front side of Follower (Trailer) robot at an angle of ϑ.
T_L_ ϑ_	Truck Robot (Leader) at an angle of ϑ.
T_F _ ϑ_	Trailer Robot (Follower) at an angle of ϑ.
S_LF_ ϑ_	Front sensor of leader (Truck) robot at an angle of ϑ.
S_LL_ ϑ_	Left sensor of leader (Truck) robot at an angle of ϑ.
S_LR_ ϑ_	Right sensor of leader (Truck) robot at an angle of ϑ.
